# Avian Pathogenic *Escherichia coli* T6SS Effector Protein Hcp2 Induces Mitochondrial Dysfunction and Activates Mitophagy in Chicken Tracheal Mucosal Epithelial Cells

**DOI:** 10.1155/tbed/4882962

**Published:** 2026-06-25

**Authors:** Bingyu Zhao, Ziqi Li, Liyang Dai, Yifei Huang, Yaqin Tian, Xiyang Wei, Chenchen Sheng, Zhenyu Wang, Ying Shao, Jiumeng Sun, Jian Tu, Xiangjun Song

**Affiliations:** ^1^ Anhui Province Key Laboratory of Veterinary Pathobiology and Disease Control, College of Veterinary Medicine, Anhui Agricultural University, Hefei, 230036, China, ahau.edu.cn; ^2^ Anhui Province Engineering Laboratory for Animal Food Quality and Bio-Safety, College of Veterinary Medicine, Anhui Agricultural University, Hefei, 230036, China, ahau.edu.cn; ^3^ Joint Research Center for Food Nutrition and Health of IHM, Anhui Agricultural University, Hefei, China, ahau.edu.cn

**Keywords:** avian pathogenic *Escherichia coli*, hemolysin-coregulatory protein 2, mitochondrial dysfunction, mitophagy, type VI secretion system

## Abstract

The secretion system of avian pathogenic *Escherichia coli* (APEC) plays a key role in bacterial colonization and invasion of the host. The core structural component of the type VI secretion system (T6SS), hemolysin‐coregulatory protein (Hcp), functions both as a T6SS structural component and a secreted virulence effector. However, the pathogenic mechanisms by which Hcp2 affects host cell function remain poorly understood. In this study, we focused on the impact of Hcp2 on mitochondrial function in chicken tracheal mucosal epithelial (CTE) cells to reveal the mechanism of APEC‐induced host cell damage. Hcp2 exposure led to significant mitochondrial dysfunction, as evidenced by elevated levels of reactive oxygen species, mitochondrial membrane potential depolarization, and intracellular calcium overload. These findings suggest that Hcp2 induces mitochondrial oxidative stress and disrupts cellular homeostasis. Notably, when mitochondrial function is impaired, cells initiate a selective autophagic mechanism, a process that may be closely related to the pathogenic mechanism of Hcp2 protein. Transmission electron microscopy (TEM) and immunofluorescence microscopy confirmed the formation of double‐membraned autophagosomes. Western blot analysis further revealed increased conversion of LC3‐I to LC3‐II and a dynamic change in p62/SQSTM1 expression. Additionally, the degradation of mitochondrial proteins and the increased colocalization of mitochondria with autophagosomes and lysosomes confirmed the activation of mitophagy. Our study reveals that Hcp2 disrupts mitochondrial functional homeostasis and activates mitophagy in CTE cells.

## 1. Introduction

Avian pathogenic *Escherichia coli* (APEC) causes respiratory and systemic infections in poultry by colonizing the tracheal mucosa, resulting in high morbidity and mortality [1]. Previous studies have demonstrated that APEC infection induces significant inflammatory responses in chicken trachea and markedly alters the mRNA expression levels of genes involved in inflammatory and proliferative signaling pathways [2]. APEC possesses or utilizes different virulence and pathogenic mechanisms to cause *E. coli* disease in poultry, in which the secretion system plays a key role [3]. The type VI secretion system (T6SS), a complex nanomachine present in many Gram‐negative bacteria, has emerged as a critical determinant of pathogenicity [4]. In *Salmonella typhimurium*, T6SS is indispensable for intracellular replication and systemic transmission [5]. The hemolysin‐coregulatory protein (Hcp) participates in system assembly as a structural component of the T6SS and also performs biological functions as a virulence factor [6, 7]. Studies have shown that deletion of the *hcp* gene causes a significant defect in the virulence of *Burkholderia pseudomallei* [8]. Despite the high degree of structural homology of Hcp family proteins, their expression, regulatory mechanisms, and pathogenic effects are highly variable [9]. Hcp1 promotes inflammatory and pyroptosis in murine models to enhance *E. coli* virulence [10], while Hcp2 from *Vibrio alginolyticus* regulates flagellar morphology and expression of downstream effector genes, thereby affecting bacterial motility, adhesion, and virulence [11].

Mitochondria are essential organelles involved in energy metabolism, calcium homeostasis, and redox balance [12]. They generate adenosine triphosphate through oxidative phosphorylation to meet the energy demands of cellular metabolism and actively sequester calcium from the cytoplasm, thereby contributing to the regulation of calcium signaling [13]. Given these crucial roles, mitochondria are recognized as prime targets during microbial infections [14]. Many pathogens have developed strategies to specifically target mitochondria, altering mitochondrial dynamics and functional integrity to enhance their intracellular persistence and facilitate the evasion of host immune defenses [15]. Disruption of mitochondrial function, such as calcium overload, elevates mitochondrial reactive oxygen species (mtROS), and loss of membrane potential can result in organelle damage and cellular dysfunction [16]. It has been shown that multiple virulence factor regulators (MvfRs), regulators of *Pseudomonas aeruginosa*, significantly increase ROS levels in skeletal muscle cells, thereby triggering mitochondrial dysfunction [17]. It is worth noting that cells do not suffer damage passively. Cells activate mitophagy to eliminate damaged mitochondria, limit mtROS accumulation, and preserve cellular homeostasis [18]. For example, *Mycobacterium bovis* activates mitophagy in macrophages to evade xenophagy [19]. Whether Hcp2 induces similar mitochondrial quality control mechanisms during infection remains unknown.

Our previous studies showed that Hcp2 can localize in the mitochondria in DF‐1 cells [20, 21], suggesting that it may exert its biological function by targeting the mitochondria. In this study, we explored the impact of Hcp2 on mitochondrial function and mitophagy in chicken tracheal mucosal epithelial (CTE) cells, aiming to reveal the role of the APEC effector protein Hcp2 in host–pathogen interactions and to offer novel insights into the involvement of mitochondrial quality control in bacterial pathogenesis.

## 2. Materials and Methods

### 2.1. Cell Culture

The CTE cells used in this study were preserved by the Anhui Provincial Key Laboratory of Veterinary Pathobiology and Disease Control. Cells were cultured in Dulbecco’s modified Eagle medium/F‐12 supplemented with 10% fetal bovine serum and 1% penicillin‐streptomycin. Cells were seeded in 12‐well plates and allowed to adhere for ~12 h. When cell confluence reached about 80%, CTE cells were treated with recombinant Hcp2a and Hcp2b proteins obtained through prokaryotic expression. In the experiment, concentrations of 200 μg/mL Hcp2a and 150 μg/mL Hcp2b were used. The selected concentrations were chosen based on preliminary experiments showing evident mitochondrial functional alterations while maintaining acceptable cell viability.

### 2.2. Antibodies

The 11 antibodies used in this study were obtained from three different companies. TOM20 (11802‐1‐AP), TIM23 (11123‐1‐AP), LC3 (14600‐1‐AP), p62/SQSTM1 (18420‐1‐AP), HSP60 (66041‐1‐Ig), HRP‐conjugated goat anti‐mouse immunoglobulin G (IgG) (H + L) (SA00001‐1), and Multi‐rAb HRP‐goat anti‐rabbit recombinant secondary antibody (H + L) (SA00001‐2) were obtained from Protechtech (Wuhan, China). β‐Actin (AC026), ABflo 488‐conjugated goat anti‐rabbit IgG (H + L) (AS053), and ABflo 594‐conjugated goat anti‐mouse IgG (H + L) (AS054) were purchased from ABclonal (Boston, MA, USA). LAMP1 was purchased from Zen‐Bio (Chengdu, China).

### 2.3. Protein Purification

The recombinant plasmids pET‐28a‐hcp2a and pET‐28a‐hcp2b were preserved from the laboratory. *E. coli* BL21 (DE3) cells carrying the plasmids were cultured in Luria–Bertani broth containing 1% kanamycin. β‐D‐1‐thiogalactopyranoside (IPTG) (Sangon Biotech, Shanghai, China) at a concentration of 0.25 mM was used to induce the expression of the Hcp2a protein, while 1 mM IPTG was used for Hcp2b induction. Protein expression was performed at 16°C for 16 h to enhance the yield. The supernatant from lysed bacteria was incubated with HisSep Ni‐NTA MagBeads (20561ES08; Yeasen Biotechnology, Shanghai, China). Finally, proteins were sequentially eluted using buffers containing 40 and 250 mM imidazole. Finally, the BCA protein assay kit (EC0001‐A; Sparkjade, Shandong, China) was used to determine the Hcp2 protein concentration after endotoxin removal (L00338; GenScript, Nanjing, China).

### 2.4. Mitochondrial Calcium Measurement

Mitochondrial calcium levels were determined using Rhod‐2, AM, a mitochondrial calcium‐specific fluorescent probe (MX4507; Maokang Biotechnology, Shanghai, China). Cells were treated with Hcp2a or Hcp2b protein for 6 h. Rhod‐2, AM working solution was prepared according to the manufacturer’s instructions and incubated with cells at 37°C for 20 min. After washing with Hanks’ buffer with 20 mM HEPES, cells were incubated for an additional 30 min at room temperature to ensure complete deesterification. Finally, fluorescence was detected using an inverted fluorescence microscope.

### 2.5. Mitochondrial Superoxide Detection

Mitochondrial ROS levels were assessed using the MitoSOX Red fluorescent probe (S0061S; Beyotime Biotechnology, Shanghai, China), which selectively detects superoxide in mitochondria. CTE cells were treated with Hcp2a or Hcp2b protein for 6 h. Cells were then washed with phosphate‐buffered saline (PBS) and stained with MitoSOX Red working solution. For the positive control, cells were preincubated with the kit‐provided mSoxUp (1×) for 4 h to induce superoxide production. Untreated cells served as the negative control. After 30 min of staining at 37°C, the excess dye was removed with PBS. Finally, fluorescence imaging was performed using an inverted fluorescence microscope.

### 2.6. Mitochondrial Membrane Potential Assay

Mitochondrial membrane potential (Δψm) was analyzed using the JC‐1 assay kit (M8650; Solarbio, Beijing, China). The red‐to‐green fluorescence shift of JC‐1 indicates changes in Δψm. Cells were treated with Hcp2a or Hcp2b proteins for 6 h. After washing with PBS, cells were stained with the JC‐1 working solution. As a positive control, the CTE cells were pretreated with 10 μM of carbonyl cyanide m‐chlorophenyl hydrazone (CCCP) for 20 min before staining. Untreated cells served as the negative control. After 20 min of incubation at 37°C in the dark, the cells were washed with JC‐1 buffer (1×), and fluorescence was observed under a fluorescence microscope. JC‐1 monomers were detected in the green fluorescence channel (Ex/Em = 488/530 nm), whereas JC‐1 aggregates were detected in the red fluorescence channel (Ex/Em = 561/590 nm).

### 2.7. Immunofluorescence Assay

The CTE cells were treated with Hcp2a or Hcp2b proteins for 6 h. They were then fixed with 4% paraformaldehyde for 15 min, permeabilized with 0.1% Triton X‐100 for 10 min, and blocked with 5% skim milk for 1 h at room temperature. After overnight incubation with primary antibodies at 4°C, the cells were stained with fluorescent secondary antibodies for 1 h at room temperature. The nuclei were stained with 4^′^, 6‐diamidino‐2‐phenylindole for 10 min. For mitochondrial labeling, MitoTracker Red CMXRos (C1035; Beyotime Biotechnology, Shanghai, China) was used to stain mitochondria in live cells before fixation. Cells were imaged with a Leica confocal microscope.

### 2.8. Transmission Electron Microscopy (TEM)

The CTE cells grown in 10‐mm dishes were treated with Hcp2a or Hcp2b proteins. Untreated cells served as negative controls. After 6 h, the cells were washed with PBS and collected via centrifugation (1000 rpm for 3 min), after which they were fixed in Gluta fixative (P1126; Solarbio, Beijing, China) for 24 h at 4°C. Subsequent embedding, sectioning, and imaging were performed by the Sci‐go Instrument Testing Platform.

### 2.9. Western Blot Analysis

For immunoblot analysis, treated cells were lysed in radioimmunoprecipitation assay buffer supplemented with a 1% protease inhibitor for 5 min on ice. The total protein was separated via sodium dodecyl sulfate‐polyacrylamide gel electrophoresis and then transferred onto polyvinylidene fluoride membranes. The membranes were blocked in 5% skim milk and incubated overnight at 4°C with primary antibodies and subsequently with HRP‐conjugated secondary antibodies for 1 h at room temperature. The protein bands were visualized using an enhanced chemiluminescence system (SQ203; Epizyme Biotech, Shanghai, China). Band intensity was quantified using ImageJ software, and statistical analysis was conducted using GraphPad Prism software.

### 2.10. Statistical Analysis

All data were analyzed using GraphPad Prism 8.0 software. A one‐way analysis of variance was used for comparisons among multiple groups. Tukey’s post hoc test was further applied for pairwise comparisons among all groups.

## 3. Results

### 3.1. Hcp2 Protein Increases Mitochondrial Calcium Concentration in CTE Cells

**Figure 1:**
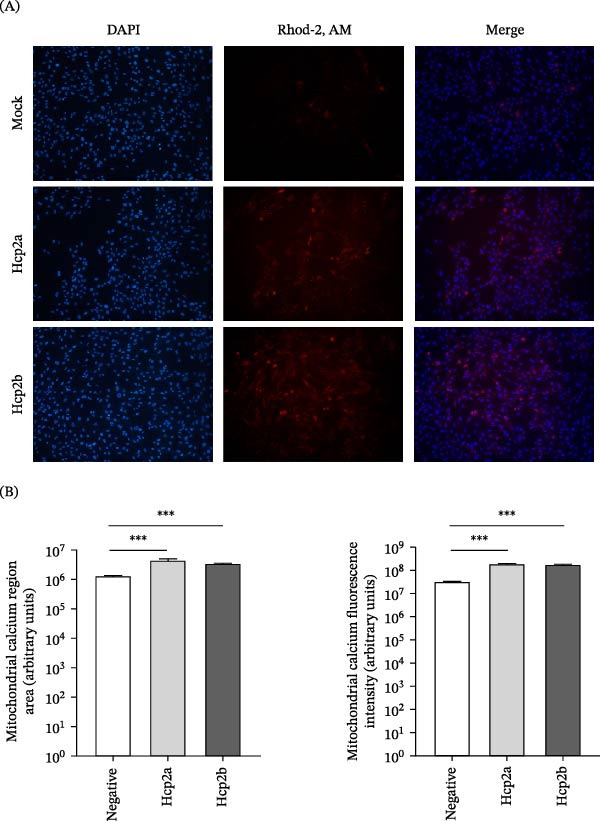
Hcp2 protein increases mitochondrial calcium concentration in chicken tracheal mucosal epithelial cells. (A) Cells exposed to Hcp2 exhibited significantly increased Rhod‐2 fluorescence area and expanded fluorescent intensity; control cells show only a weak mitochondrial Ca^2+^ signal. Scale bar = 50 µm. (B) Analysis of fluorescence area and fluorescence intensity by ImageJ. Mitochondrial Ca^2+^ levels in the Hcp2‐treated group significant increased compared to the control. Fluorescence intensity and area were obtained from three independent replicate experiments, with results expressed as mean ± SD.  ^∗∗∗^ = Significantly different (*p* < 0.001).

After 6 h of Hcp2 protein treatment, fluorescence imaging revealed that fluorescence intensity and the area of fluorescence coverage were significantly (*p* < 0.001) increased compared to the untreated control group (Figure 1). These findings indicated that the Hcp2 protein induces calcium overload in the mitochondrial matrix, thereby disrupting calcium homeostasis in CTE cells.

### 3.2. Hcp2 Protein Increases Mitochondrial Superoxide Levels in CTE Cells

**Figure 2:**
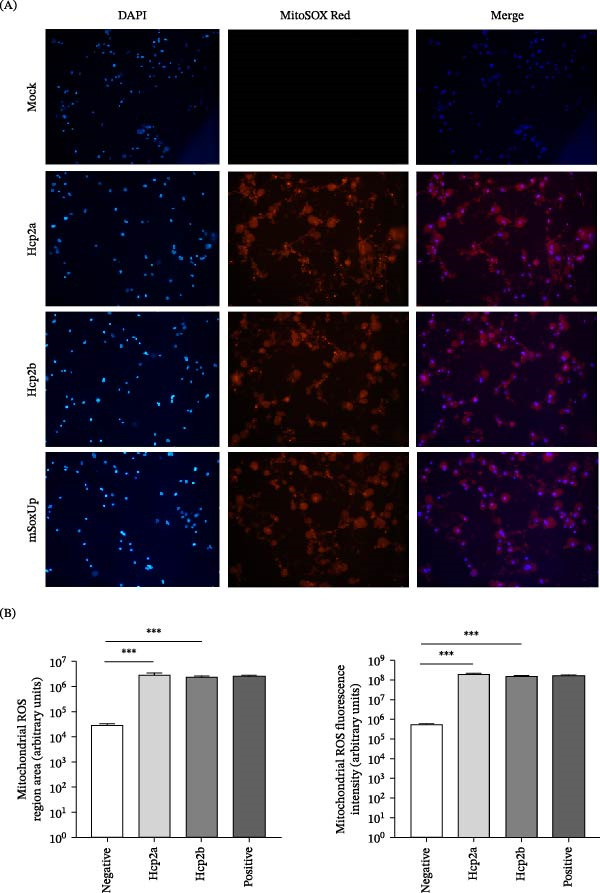
Hcp2 protein increases mitochondrial superoxide levels in chicken tracheal mucosal epithelial cells. (A) Confocal microscopy of Hcp2‐treated cells exhibited markedly enhanced MitoSOX fluorescence intensity and increased area of fluorescence distribution compared to the untreated control group. The mtROS‐inducing effect of Hcp2 was comparable to that observed in the mSoxUP‐positive control group. Scale bar = 50 µm. (B) Quantitative analysis of fluorescence area and fluorescence intensity showed a significant increase in mtROS levels in the Hcp2‐treated group. Fluorescence intensity and area were obtained from three independent replicate experiments, with results expressed as mean ± SD.  ^∗∗∗^ = Significantly different (*p* < 0.001).

Compared with the control, CTEs exposed to Hcp2 protein for 6 h exhibited significantly (*p* < 0.001) increased mtROS fluorescence intensity and an expanded fluorescence‐positive area (Figure 2). Notably, this mtROS enhancement is comparable to the trend observed in the mSoxUp‐treated group, suggesting that the Hcp2 protein triggered aberrant superoxide accumulation within the mitochondrial matrix.

### 3.3. Hcp2 Protein Decreases Mitochondrial Membrane Potential in CTE Cells

**Figure 3:**
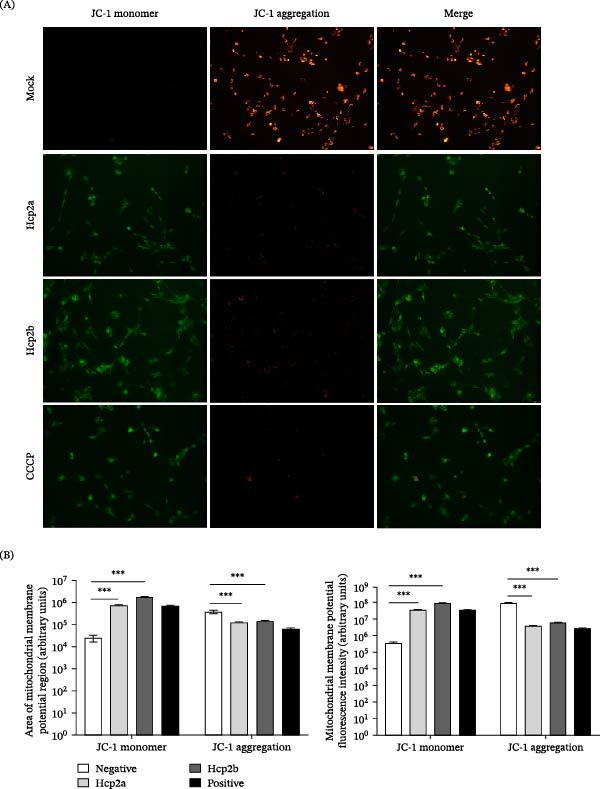
Hcp2 protein decreases mitochondrial membrane potential (Δψm) in chicken tracheal mucosal epithelial cells. (A) In control cells with intact Δψm, JC‐1 aggregates emitted red fluorescence, whereas in cells with depolarized mitochondria, JC‐1 existed mainly as monomers emitting green fluorescence. Confocal imaging showed that Hcp2‐treated cells exhibited reduced red fluorescence and increased green fluorescence similar to the carbonyl cyanide m‐chlorophenyl hydrazone‐treated group, indicating significant mitochondrial membrane depolarization. Scale bar = 50 μm. (B) Analysis of fluorescence area and fluorescence intensity by ImageJ. The ratio of red/green fluorescence intensity was significantly decreased in the Hcp2‐treated group compared with the control. Fluorescence intensity and area were obtained from three independent replicate experiments, with results expressed as mean ± SD.  ^∗∗∗^ = Significantly different (*p* < 0.001).

Following the 6‐h exposure to Hcp2 protein, fluorescence analysis demonstrated that the red fluorescence (JC‐1 aggregates) significantly (*p* < 0.001) declined, while green fluorescence (JC‐1 monomers) was significantly (*p* < 0.001) elevated (Figure 3). These results closely mirrored those of the CCCP‐treated group, indicating that Hcp2 protein induced mitochondrial depolarization and compromised membrane potential integrity.

### 3.4. Hcp2 Protein Induces Mitochondrial Fragmentation in CTE Cells

**Figure 4:**
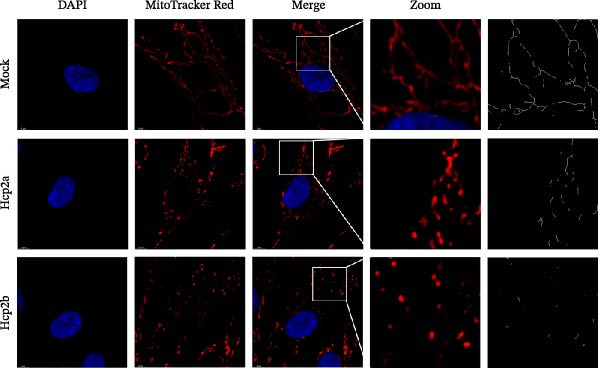
Hcp2 protein induces mitochondrial fragmentation in chicken tracheal mucosal epithelial cells. In control cells, mitochondria displayed an elongated, tubular network typical of healthy cells. Cells exposed to Hcp2 exhibited a marked shift to fragmented, punctate mitochondrial structures, indicating disruption of mitochondrial dynamics. Scale bar = 5 µm.

Compared to the untreated control group, the mitochondria in the Hcp2‐treated cells exhibited a marked shift from an extended, interconnected network to a fragmented morphology, characterized by shorter, punctate mitochondrial structures (Figure 4). This disruption of the mitochondrial network suggests that the Hcp2 protein induced mitochondrial fragmentation, potentially reflecting an imbalance in mitochondrial fission and fusion dynamics or an early step in mitophagy activation.

### 3.5. Hcp2 Protein Promotes Autophagosome Formation in CTE Cells

**Figure 5:**
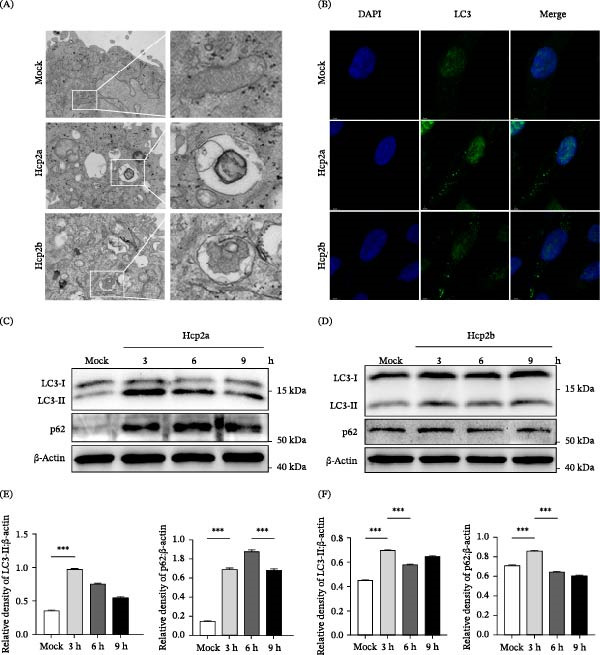
Hcp2 protein promotes autophagosome formation in chicken tracheal mucosal epithelial cells. (A) Typical double‐membrane autophagosomes in cells treated with Hcp2a or Hcp2b for 6 h. (B) Increased LC3 puncta formation in Hcp2‐treated cells, indicating autophagosome accumulation. Scale bar = 5 µm. (C) Western blot analysis of autophagy‐related proteins LC3 and p62 in cells treated with Hcp2a protein for four different durations. (D) Quantification of LC3‐II and p62 band intensities using ImageJ. (E) Western blot analysis of LC3 and p62 in cells treated with Hcp2b protein. (F) Quantification of LC3‐II and p62 band intensities using ImageJ. Data in (D) and (F) are presented as mean ± SEM from three independent experiments. Relative protein levels were normalized to β‐actin.  ^∗∗∗^ = Significantly different (*p* < 0.001).

The CTE cells treated with the Hcp2 protein exhibited characteristic double‐ or single‐membrane autophagosomes containing cytoplasmic materials to be degraded (Figure 5A), suggesting that the cell underwent an active autophagic process. These structures were absent in the untreated control group. Additionally, fluorescence microscopy revealed markedly increased and aggregated LC3‐positive puncta in the Hcp2‐treated cells (Figure 5B), further supporting the potential role of the Hcp2 protein in promoting autophagy. Changes in key autophagic proteins can provide stronger evidence that autophagy occurs in cells. Western blot analysis showed a significant (*p* < 0.001) increase in LC3‐II levels, indicative of enhanced autophagosome formation. Meanwhile, the autophagy substrate p62/SQSTM1 exhibited a dynamic pattern of initial accumulation followed by degradation (*p* < 0.001), suggesting that the autophagic flux may undergo a dynamic change, with early accumulation and late degradation of p62/SQSTM1 (Figure 5C–F).

### 3.6. Hcp2 Protein Induces Mitophagy in CTE Cells

TEM analysis revealed the presence of typical mitophagic structures in Hcp2‐treated cells, characterized by swollen mitochondria with disrupted cristae enclosed within double‐membrane autophagosomes, which are recognized as hallmark features of mitophagy. In contrast, mitochondria in the untreated group retained intact morphology and well‐defined cristae (Figure 6A), indicating that the Hcp2 protein triggered mitophagy. Western blot analysis revealed a significant (*p* < 0.001) reduction in the expression of mitochondrial marker proteins (TOM20 and TIM23) and matrix protein HSP60 following Hcp2 treatment (Figure 6B–E). Additionally, we observed increased colocalization of mitochondria with autophagosomes and lysosomes in the Hcp2 group (Figure 6F–G), suggesting that dysfunctional mitochondria were ultimately delivered to lysosomes for degradation. Together, these results demonstrate that the APEC effector Hcp2‐induced mitophagy in CTE cells, resulting in the targeted removal of damaged mitochondria through the autophagy‐lysosome system.

Figure 6Hcp2 protein induces mitophagy in CTE cells. (A) Characteristic mitophagosomes in cells treated with Hcp2a or Hcp2b for 6 h. Scale bar = 0.5 μm. (B) Western blot analysis demonstrated decreased expression of mitochondrial proteins (HSP60, TOM20, and TIM23) in cells treated with Hcp2a. (C) Western blot analysis of mitochondrial proteins (HSP60, TOM20, and TIM23) in cells treated with Hcp2b. (D) Densitometric quantification using ImageJ revealed a significant reduction in mitochondrial proteins levels compared to the control in cells treated with Hcp2a. (E) Densitometric quantification using ImageJ revealed a significant reduction in mitochondrial proteins levels compared to the control in cells treated with Hcp2b. Data in (D) and (E) are presented as mean ± SEM from three independent experiments. Relative protein levels were normalized to β‐actin.  ^∗^ = Significantly different(*p* < 0.05).  ^∗∗∗^ = Significantly different (*p* < 0.001). (F, G) Confocal imaging demonstrated enhanced colocalization of mitochondria with autophagosomes and lysosomes, indicating the delivery of mitophagosomes to lysosomes for degradation. Colocalization levels were quantified using Pearson’s correlation coefficient, with data expressed as mean ± SD. Data were compared via one‐way ANOVA with Tukey’s post hoc test. Scale bar = 5 μm.

**Figure:**
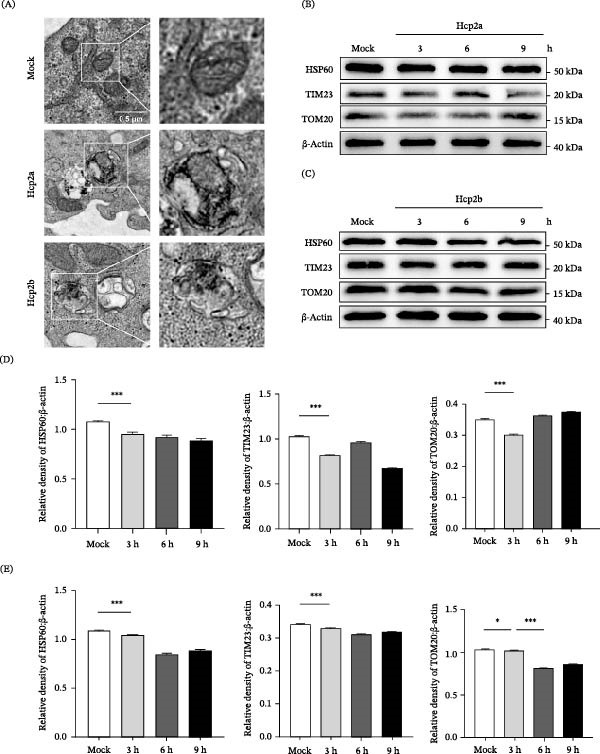

**Figure:**
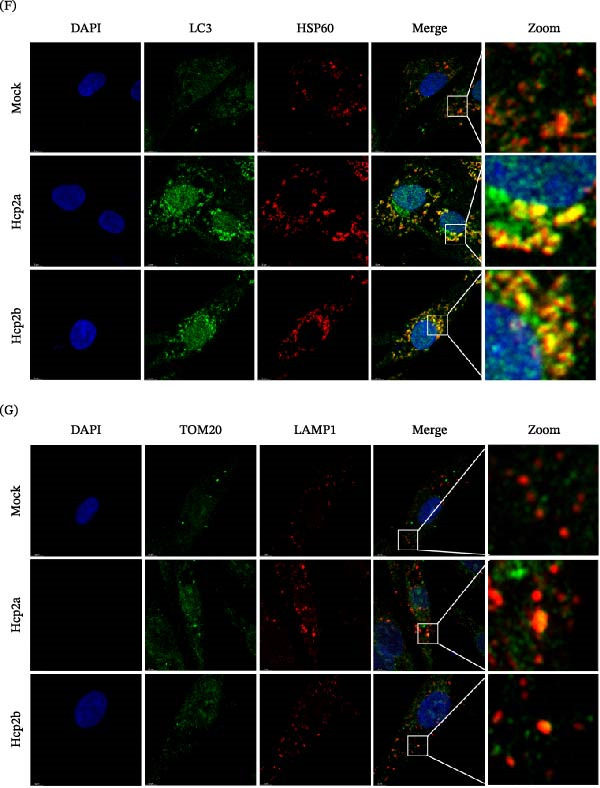

## 4. Discussion

The T6SS is a key virulence determinant of APEC, enabling the delivery of effector proteins such as Hcp into host cells to facilitate competitive survival [22]. In the APEC T6SS, Hcp2a and Hcp2b are paralogous proteins encoded by distinct genes within the same T6SS gene cluster. Their structural function as T6SS channel components is conserved, but they have diverged in sequence and may possess distinct functions [23, 24]. Members of the Hcp protein family have been implicated in modulating host immune responses and cell death pathways [7, 10], but the specific role of Hcp2 in regulating mitochondrial dynamics and selective autophagy remains poorly understood. Given the central role of mitochondria in innate immunity and cellular stress responses, we investigated whether Hcp2 could induce mitochondrial dysfunction and trigger mitophagy in CTE cells.

Our results demonstrated that the APEC effector Hcp2‐induced significant mitochondrial dysfunction, as evidenced by elevated intracellular calcium levels, increased mtROS, and a significant decrease in Δψm. These three revelations are widely recognized as hallmark indicators of mitochondrial stress [12]. The disruption of intracellular calcium homeostasis is usually one of the key steps in response to pathogenic stimuli [25]. Mitochondrial calcium overload can form in the contact region between mitochondria and Ca^2+^ channels of the endoplasmic reticulum (ER) [26]. Excessive mitochondrial Ca^2+^ augments tricarboxylic acid cycle dehydrogenase activity, enhancing electron transport chain function and thereby promoting excessive ROS generation and oxidative damage [27]. Simultaneously, ROS‐induced oxidative stress damages mitochondrial lipids, proteins, and mtDNA, further impairing mitochondrial function and amplifying Δψm depolarization [28]. Calcium overload, ROS accumulation, and loss of Δψm together constitute the major drivers of mitochondrial dysfunction during host–pathogen interactions.

Consistent with the observed mitochondrial dysfunction, we also found that treatment with the Hcp2 protein led to significant morphological alterations in the mitochondrial network. In contrast to the elongated and interconnected tubular structures in the control cells, mitochondria in the Hcp2‐treated CTE cells exhibited a fragmented phenotype, characterized by shortened punctate organelles. This mitochondrial fragmentation suggests that Hcp2 disrupts the balance between mitochondrial fission and fusion. Fragmentation of the mitochondria is often associated with mitochondrial stress and is considered a hallmark of early mitophagic processes or apoptotic signaling pathways [29]. Therefore, the structural remodeling of the mitochondrial network may represent an adaptive cellular response to Hcp2‐induced stress, likely contributing to the initiation of mitophagy for the removal of damaged organelles.

Mitochondrial damage thus appears to be an early and central event in the host response to Hcp2 exposure. In response to this injury, the autophagic response is efficiently activated. TEM revealed double‐membrane autophagosomes, while immunofluorescence microscopy confirmed LC3 puncta formation. Western blot analysis showed an increase in LC3‐II levels, indicating enhanced autophagic flux. Interestingly, p62/SQSTM1 levels exhibited a biphasic pattern—initially elevated and then subsequently decreased. This dynamic regulation of p62/SQSTM1 suggests that Hcp2 may first stimulate autophagosome formation via inhibition of the mTOR signaling pathway or by activating adenosine monophosphate‐activated protein kinase, both of which are well‐characterized, upstream regulators of autophagy initiation [30]. The subsequent clearance of both p62/SQSTM1 and LC3‐II implies that the autophagosome effectively fuses with the lysosome to complete the autophagic process.

Notably, changes in mitochondrial functional indicators not only signify organelle damage but also actively contribute to the initiation of mitophagy. It is well known that mitochondrial membrane depolarization stabilizes PTEN‐induced kinase 1 on the outer mitochondrial membrane, which in turn recruits the E3 ubiquitin ligase Parkin [31]. Parkin mediates the ubiquitination of outer mitochondrial membrane proteins (e.g., TOM20) and facilitates their recognition by the autophagic machinery [32]. In our study, multiple lines of evidence support the activation of mitophagy following Hcp2 treatment: the presence of double‐membraned mitophagosomes observed by TEM; the marked downregulation of mitochondrial proteins; and the increased colocalization of mitochondria with autophagosomes and lysosomes, as revealed by confocal microscopy.

## 5. Conclusions

Our findings demonstrate that Hcp2, the T6SS effector protein of APEC, induces mitochondrial dysfunction in CTE cells, which in turn activates both general autophagy and selective mitophagy. This process is driven by a cascade involving mitochondrial Ca^2+^ overload, oxidative stress, and Δψm loss, which ultimately leads to autophagosome formation and clearance of damaged mitochondria. This sequential response emphasizes the ability of host cells to initiate stress‐adaptive mechanisms to maintain intracellular homeostasis in the face of bacterial virulence.

## Author Contributions

**Bingyu Zhao**: data extraction, data curation, analysis and interpretation of data, data visualization, writing – original draft, writing – review and editing. **Ziqi Li**: data extraction, data curation, analysis and interpretation of data. **Liyang Dai, Yifei Huang, Yaqin Tian, Chenchen Sheng, Zhenyu Wang, Ying Shao, and Jiumeng Sun**: data curation, formal analysis. **Xiyang Wei**: data extraction, data curation. **Jian Tu**: conceptualization, investigation, methodology. **Xiangjun Song**: project administration, funding acquisition, resources, conceptualization, methodology, supervision.

## Funding

This work was supported by grants from the Natural Science Foundation of China (Grant 32272959), the Anhui Provincial University Excellent Young Talents Support Program (Grant YQYB2023001), and Research Funds of Joint Research Center for Food Nutrition and Health of IHM (Grant 2024SJY04).

## Ethics Statement

The chicken tracheal mucosal epithelial cells used in this study are maintained by the Anhui Provincial Key Laboratory of Veterinary Pathobiology and Disease Control at Anhui Agricultural University. All cells have been tested for mycoplasma to ensure that they are free of contamination. They are obtained from legitimate sources and comply with academic ethical standards.

## Conflicts of Interest

The authors declare no conflicts of interest.

## Data Availability

The data that support the findings of this study are available from the corresponding author upon reasonable request.
